# Editorial

**Published:** 1856-12

**Authors:** 


### EDITORIAL DEPARTMENT.

### MEDICAL JOURNALS----THEIR CLAIMS TO SUPPORT.

Is there an individual in the profession, who is interested in its well-being, or who is imbued with a desire for scientific improvement and advancement, who will now ask himself the question whether the Journals of the country have contributed to these ends? He may properly indulge in speculations with reference to the extent of their influence for good, and also into the manner and matter somewhat, but he will never, not even for a moment, entertain a doubt as to the efficiency or the power of our periodical literature over the medical mind of the country. There is not one, we will venture the declaration, who claims a respectable standing among his professional brethren, who will not raise his voice in favor of the journalism of our land, because of the substantial benefits it has bestowed upon, and diffused through and among, the medical profession.

There are two classes, who, by *words* and *acts*, affect to sneer at their inefficiency and incompetency, and decry them as worthless trash and useless rubbish, and these are composed, in the first place, of those who never subscribed for one, and therefore as they never examined into their contents, and were, therefore, eminently calculated to judge of their merits; and those who, having subscribed, perused them, filed them away, but whose minds and memories were so pre-occupied and engrossed with the things of the world, that they neglected payment altogether. This is a very liberal apology, and infinitely more so than we really entertain for the latter. Now this class, unfortunately for the publishers, are the most numerous. They may not rail out openly, may not give expression to a single word of disapproval, may read and re-read the pages, bestow them away upon the shelf, and even look upon each number with complacent expressions upon their countenances. They have given their names to the publisher, they have been placed upon the list, and this magnanimous act, they appear to think, is worth much to those who are laboring assiduously for their benefit and improvement. There is their sign-manual, which is equivalent to the broad smiles of their countenances, they have patronised the work, for they are "*subscribers.*" This is enough, the *material aid* must come from those in whose names there is less potency.

It is of no moment to them that the editors have wasted away the hours which have been set apart for their repose, demanded alike for their comfort, well-being, and health. It matters little how much of responsibility and care have weighed upon their minds, touching the *matter* and the *manner* of its pages. It is of but small concern what may constitute the outlays and expenses of the projectors and proprietors, or how much of the "bread" may again be gathered which has been freely "cast upon the waters." It is wholly unobserved and unthought of how many hours of close labor the compositors, pressmen, and binders have bestowed upon the work, or whether they have been rewarded for their honest toil. The work comes regularly, laden with its stores, and this is their *only* concern, and the ultimatum of their thoughts or cares.

How unjust and preposterous to ask so much as a gratuitous bestowment. How unequal, as well as unfair, to reward others for the most trivial service and leave those unpaid who are laboring daily and hourly in order to furnish them regularly with their mental pabulum.

It is to these, then, and not to those who eschew all Journals, that we would address ourselves, as those who are especially a withering blight upon the process of the periodical literature of the country. And we would address them plainly, that each may see the position he occupies, and in order to this, we would propound a few plain practical interrogatories: Do you desire to see all new scientific discoveries and facts, and all fresh medical intelligence promptly laid before, and freely diffused throughout the profession? If so, what medium would you select for this purpose and end, which you are prepared to admit is so essential? In the absence of periodicals, what means would you adopt if you were desirous either of communicating some new and valuable discovery in medical science, or of being informed, as early as possible, of that of some other member of the profession? Perhaps a deadly epidemic sweeps through the land, leaving behind it the dread evidences of its powers, and in your hands, indeed your own discovery in the treatment of the fatal malady, has proved very efficient in its control. In this case promptness and expedition are required in order that the demands of humanity may be answered, and yet, there is no channel through and by which you can reach your medical brethren. The secular publications will not avail, for in the time of distress, when the public health is suffering, the medical man has no time to read, except to make occasional references. There is no time to write, print, or publish a book, for the occasion which demanded the requisite and valuable information has perhaps passed away.

In this presumed extremity, where would you look for the best, the surest, and most prompt mode of diffusion and distribution? To the Medical Journals and to *no other* means, because they have proved competent in the past, is the proper channel in the present, and the hope in the future; and which, too, at this time, are so numerous that there is one for almost every week in the year, and are daily flying in our mails from the East, West, North, and South, laden with medical intelligence, and furnishing, in the aggregate, a vast fund of sedate and interesting facts, to be quickly and freely diffused throughout the length and breadth of the land. And yet we are well assured that the majority of those who are thus catering for the public good, are contributing so much in the cause of humanity, who are elevating the standard of the profession, and are collecting such a large fund to be added to the stores of medical intelligence, which they themselves have already elicited, do not receive *one farthing for their labors.* Few of our Journals are profitable, a few may sustain themselves, while others again, who are in their struggling infancy, are unable to walk alone, but must be assisted. This is precisely the situation of this Journal, for its parents are compelled to pay out of their own pockets for its support.

We are not writing an appeal to the magnanimity and generosity of the members of the profession. Their own superior intelligence will enable such to perceive the true position of a majority of the Journals now issued, when the truth is suggested. It is intended to arouse the indifferent to the discharge of a duty they owe to proprietors and publishers of these Journals, to themselves and the profession. There are a few magnanimous members of the profession, who, at whatever sacrifice, will not allow the Journals to go down, but these should not be required to bear the burthen, however willing they may be to do so. In this State, and among our subscribers, there are some names which it will be a pleasure for us to refer to hereafter, whose liberality and spirit, in sustaining a medical journal in a new State, deserve all praise, and it shall be publicly awarded to them when we come to write its history. No, we are making no appeal for ourselves, but are earnestly alive to the best interests of our medical brethren around us. It is not ours, it is theirs; it is not for us, it is for themselves, that we are peculiarly interested; it is not for the Journal, it is for the advancement of the profession in science and knowledge, in which they are equally interested. Our position is only one of trust, and we would not discharge the obligation, unless we can secure the support, the favor, and endorsement of those who have, in generous confidence, confided that duty to our management, and its performance to our capacity and judgment.

Let, then, each one discharge his trust. An obligation rests, with *great weight*, upon each professional man. He is bound by the promises he has made when he received his degree, and by his public announcement of his position in society, to do all in his power for the advancement of medical science, and he will be accounted as unworthy, who will show himself indifferent to the means of medical improvement. Let each subscriber make an effort to procure an additional one before the appearance of the first No. of Vol. IV, and as many more as is possible for him to procure.

---

We cheerfully give place to the following circular of our esteemed friend, Dr. Gordon, and we hope, as we confidently believe, that many valuable facts will be developed through his well directed and energetic efforts in relation to the disease, the nature of which now engages his attention and employs his time and talents. We trust, too, that his circular will meet a prompt response from those whose familiarity with the disease would give their opinions weight and entitle them to consideration. Because of his previous researches into the nature and treatment of Cholera, the appointment of Dr. Gordon, as Chairman of the Committee on that subject, was a fortunate one.

TO THE MEDICAL PROFESSION AND SCIENTIFIC OBSERVERS.

The undersigned having been appointed, at the late meeting of the American Medical Association, Chairman of the Committee on the Etiology and Pathology of Epidemic Cholera, respectfully solicits Etiological, Pathological, and Historical data pertaining to the disease, from the Medical profession; and Meteorological data from any person who may have them.

As a primary examination in investigating the cause of Asiatic Cholera, and its mode of travel, he wishes to be able to compare Meteorological data obtained when the disease was raging in any given locality, with those which were obtained when the disease did not prevail.

To make this comparison most complete, a full set of Barometrical, Thermometrical, Hygrometrical, Ozonometrical tables, with prevailing winds—and Electrical pheuomena as far as obtainable—for a series of years, embracing those of health and disease, and extending over a large territory, are needed; but where full tables are not obtainable, partial ones, or reports, will be thankfully received, if the observations are accurate.

As Hygrometrical observations are extremely rare in the United States, the undersigned hopes that each person who has any which may be of value in the present investigation will forward them to his address, or inform him of their existence, and how they may be made available.

It is also hoped that every person properly prepared to keep Meteorological Registers, will spare no pains to make them as perfect as possible, and not fail to embrace Hygrometrical observations in their tables.

Tables now forming may be of real service to the Committee before the time expires for which it was appointed.

All data received prior to March 15th, 1859, will receive the attention of the Committee, and due credit be given in every instance for valuable information.

The subjects of investigation are of such vast interest to mankind, it is hoped the present request will elicit the hearty co-operation of the Medical Profession, and any other persons who may have, or shall hereafter collect matter of the kind requested.

It would seem that he should not hope in vain, when it is considered that Cholera is a disease which knows no political bounds, and frequently appears to regard neither geographical lines or sanitary cordons, and has already destroyed nearly, if not altogether, one hundred millions (100,000,000) of the earth's inhabitants.

Thus far, all with whom the Chairman has corresponded have most promptly proffered any assistance in their power, and at the same time expressed their warmest sympathy and regard for the investigation; but believing those with whom he is unacquainted may have data of the kind sought, he takes the present mode of presenting his wishes; hoping they may be complied with by all who are possessed of such data, and feel an interest in man's emancipation from disease.

Medical Journals, Literary Magazines, and Newspapers, friendly to the great interests of humanity and Medical Science, will confer a favor upon the Committee—and, it is hoped, upon the world—by inserting the above in their columns.

T. Winslow Gordon, M. D.

Georgetown, Brown Co., O., Oct., 1856.

---

### DENTISTRY.

Perhaps few arts have been more progressive than practical dentistry. A degree of perfection in the insertion of teeth, &c., has been reached which is truly surprising. Indeed, it seems now that some prefer the duplicates to the original, as the former will not *ache*, unless we are to believe the man who insists that some teeth inserted by Dr. R. S. Barber, of this city, for him were *so natural* that they did give him a few nervous twangs, whenever he forgot, as at first he often did, that they were artificial.

These remarks have been suggested by observing several specimens of work done by Dr. Barber, amongst them the substitution of an artificial structure where a large portion of the upper maxillary bone, with its teeth and palate, had been removed by a surgical operation. The whole aspect of the face was thereby restored, the voice, which had been much affected, regained its power, and the teeth performed their usual functions with comfort. Another, where the Areolæ of both the upper and lower jaws had been removed by disease, to restore which, with comfort to the patient, several distinguished dentists had spent, in vain, their skill, yet he was completely successful. To a great natural mechanical talent, the Doctor has added the science of his department, and bids fair to excel greatly in his profession.

---

### ECRASEUR.

The above is a new instrument, invented by Chassaignac, which consists of a jointed wire resembling the chain saw, but without serrated edge. This passes through a flattened canula, being attached (each end) to a shaft which slides in the canula and are moved by a handle at the other end. The instrument is for the strangulation and removal of tumors, and intended to supercede the ligature. The jointed wire passes over the tumor in a loop, when, by pulling upon the handle of the shafts, the wire is drawn tightly upon the base of the tumor.

An article in the "New York Journal of Medicine," now lying before us, details a case in which its usefulness was made very manifest, in the removal of a painful hemorrhiodal tumor, which had defied all other means, and this was effected without scarcely the loss of a single drop of blood. This instrument promises to become a most valuable accession to the appliances of the Surgeon.

---

### PRESENTATION OF A GOLD-HEADED CANE TO PROF. MEEKER, BY THE CLASS.

As a testimony of the respect and affection, by the Class, for the faithfulness and ability exhibited by Prof. Meeker, in the discharge of the duties connected with his department, the students of the Class of this session procured an elegant gold-headed cane, with an appropriate inscription, which was presented by Mr. Leas in presence of the Class, accompanied with an eloquent and appropriate address, which was replied to by Prof. Meeker, both which we give below. By resolution of the Class, the proceedings were tendered us for publication, and we cheerfully give them a place in our Journal. EDS.

THE ADDRESS.

PROF. MEEKER:—The Class of 1856–57 of the Medical Department of the University of Iowa, highly appreciate your services as their teacher; they desire to offer you some public and enduring token of their sense of obligation for your labors in their behalf—which have been constant and arduous, and generously extended by you even beyond the ordinary routine of duties imposed by the University upon the incumbent of your professional chair. It would be sufficient for them to thank you in language only, and commit their expressions of regard to the keeping of unaided memory alone, if they be satisfied to be merged with the many who have enjoyed the privilege of social and professional intercourse with you in past years, and receive only that portion of remembrance which would then legitimately belong to them.

The gentlemen of the class, however, aspire to more than this, and after repeated attempts so to diplomatize with the years of time and miles of distance, which will soon begin to separate you from them, as to secure for themselves the largest possible share of your friendly remembrance; they determined to ask you to carry about your person the slight token of their regard, which it is now my agreeable office to present. It has but little intrinsic value, but it is a gift suited to be frequently with you, especially as a companion in your walks, which your profession often requires to be solitary missions of mercy—and at such times it will suggest the names and faces of those who are now before you, and serve to remind you, that though personally absent, their gratitude, respect, and best wishes, for your health and happiness, are forever with you.

It is no slight matter for one, no longer in the youthful and athletic years of life, and in the ample enjoyment of the rewards of long and laborious professional service, to absent himself from those having social and professional claims upon him at home, and come,—in the midst of winter—with the prospect of considerable exposure awaiting him, and day after day, discharge double duties in the lecture room, and when the working hours of the week are ended, still remain, ready to give his time and valuable instruction to those who seek it, and that too, with advice so lucid and profound, and with manners so social and cordial, that those who came at first only as students, came ever afterwards in the double relation of student and friend.

It is no small matter, also, that the vigorous and flourishing young school of medicine, to which we are hereafter to refer as the guarantee of our claims to public confidence, should have enrolled among her Faculty, one whose experience as a teacher, and whose name as a Surgeon and Anatomist, not only has no superior in the West, but is familiarly known in all sections of our country. It is an evidence, to those who may be contemplating medical studies, of the growing influence and dignity of this branch of the University of Iowa, and it will enable those of us who compose the present class, when asked for an endorsement of our competency, to point with pride and confidence to the name of our Alma Mater.

The slight token we ask you to accept is a walking-stick, bearing a superscription which will preserve forever before your memory the date, the individuals, and the associations of this occasion. It is given with the hope that it may be your companion along no rough or painful path in life, because it is hoped none such shall ever be traversed by you. That it may be with you at no scene of sorrow or misfortune—if such be possible—or if that hope be too fond—and as seems to be the lot of all who make the pilgrimage of life—if the pilgrim's head must sometimes bow with sorrow, and the foot be sometimes sore and weary—that it may serve you to lean upon awhile and rest.

This place shall soon be the point from which our several paths diverge. They will be as various and wandering as our tastes and the vicissitudes in human experience can make them. Few of them can be expected to return us to this place—many may lead us to remote, and at this time, unknown fields of labor. May it so happen, nevertheless, to each of us, once and oftener before we reach the limitary line of life, that we may sometimes upon the road, look up and see a familiar form approaching us, and recognise as we near it, a learned yet modest and unassuming gentleman, our friend and former professor, D. Meeker, and discover still in his hand this cane, a gift from us in days gone by.

It is also with the wish, that it may be many a long year before it will be actually needed as a support to the person of its recipient, but that even more than the old Patriarchs, three score years and ten may be vouchsafed him, of prosperity, health, and happiness; and finally, it is given with a prayer that when at last the memories of earth must be reviewed for the last time, and all these friendly tokens laid aside—because the arm has lost its vigor and the eye its brightness—we may never hear reported to us of its owner, anything like the sad and frequent words, "he looked back along the pathway of life, and saw it strown with withered flowers," but rather as having fallen from his lips those other more glowing and eloquent words of sacred story, "when I go through the valley of the shadow of death, I will fear no evil, for thy rod and thy staff, Oh! God, they shall comfort me."

Prof. Meeker, oblige us by accepting this manifestation of our gratitude and esteem.

---

THE REPLY.

*Mr. Leas and Gentlemen of the Committee in behalf of the Medical Class:*

On this occasion permit me to return to you my most sincere thanks and heartfelt gratifications for the kindness and respect which you have manifested toward me during the present session, and more especially, for the interest you have taken in my welfare, coming among you as I did, a stranger, and having done nothing more than I felt my duty demanded of me, as a teacher, in imparting to you all the knowledge it was in my power to bestow. I have always felt it a pleasure to meet you in this hall, day after day, to elucidate, demonstrate and unravel to you the science of Anatomy. I am fully aware that you have felt the importance of the subject, from the unwearied attention which you have shown, and the rapid improvement which you have made in this department of science, during the present course. Gentlemen, in receiving this cane at your hands, which you have presented me as a further token of your generosity and respect, not only as a keepsake and relic, but as a staff to support me in my declining years, I tender you my most earnest thanks, sensible of the honor you have conferred with its presentation, and when, in coming years, I read its inscription "Presented by the Medical Class of 1856-7, of the Medical Department of the Iowa University," my mind will be brought back, in its fondest recollections, to you, Gentlemen, although distance and the mingling cares of busy life, which surround us, may widely separate us from each other. Permit me to assure you, that I most sincerely desire that I may be enabled, by an approving conscience, strict moral deportment, and professional dignity, to do honor and justice to this token of your fidelity and generosity; and my desire is that my life may be prolonged to promulgate and investigate those great truths, which belong to this department of natural science, and be able, in succeeding sessions, to meet other classes in the halls of this University, to demonstrate and delineate to them also, the beauties and mechanism of that harmonious and wonderful machine, the Human Frame.

Let me on this occasion, remind you of the duties and responsibilities which you will necessarily incur, when you enter more fully upon your professional duties. You aspire to a profession which of all is the most learned and scientific, and requires the most patient and thorough investigation to accomplish what seems to be absolutely necessary for you to understand. Communities where you may locate, will look up to you as guardians of the public health, and unless you are fully prepared to meet all reasonable demands made upon your professional services and skill, you will be like a mariner whose ship is launched into a boundless ocean, amid the storms, without a rudder to guide her safe into port. Do not let these considerations discourage you, but look back and see what has been accomplished by medical men in former ages. Many of them had not the advantages you now enjoy, but have, by perseverance and industry, left a name behind them which will be remembered as long as time shall last, never to be blotted from the pages of Medical History.

Then, Gentlemen, be not content, nor rest satisfied with what you have already attained, but continue to improve, until your name is enrolled and inscribed high on the Temple of Fame. You have a field before you to investigate and explore, a system which will require time and unintermitting mental exertions to unfold its beauties and unravel its laws, and which no other hand than that of Omnipotence could have created. Go on, then, and imitate the worthy examples of those who have so illustriously preceded you; and if you do not equal them in scientific attainment, you may at least attain that eminence in your profession which will make you an ornament to society, and a lasting benefit to suffering humanity.

After Prof. Meeker concluded, the Rev. Mr. Edwards, an old and familiar friend and patron of Prof. Meeker, was called up, and proceeded with some very feeling remarks; after which the Class called for Prof. M'Gugin, who arose and said that "he rejoiced to witness this ceremonial—this testimony of respect and affection on the part of the Class, for his esteemed colleague, one who had devoted the best energies of his life in the cultivation of medical science, and who had left his distant home, his friends, and practice to conduct the department placed under his direction and control. He trusted that the cane, presented by the Class as a testimonial of their respect for him as a man and his abilities as a teacher, would in future steady his faltering steps as he passed down on the other side of the inclined plane of life. But while this "staff would comfort him" in his physical decline, he would find great consolation in the reflection that he possessed the warm and ardent affections of the present Class.

---

### AMERICAN MEDICAL COLLEGES.

The *Peninsular Journal of Medicine* calls Dr. F. Campbell to an account for what it terms a misrepresentation by him in an address delivered in Edinburgh, not long since, on the Medical Schools and the Medical profession in the United States.

In this address, which it appears was well received, Dr. Campbell said that the Medical Schools of this country were sustained wholly by private enterprise, and that the Federal Government extended to them no patronage or protection.

Now, after an examination of the extract as furnished us, we are inclined to the belief that Dr. Campbell is not so far incorrect after all. The General Government has never yet, to our knowledge, made a direct appropriation for the establishment or maintainance of a Medical Institution. The only aid ever furnished has been indirectly bestowed, as in the grants of land to certain States for a University. This University may, or it may not, have a medical department, and yet it would not, in our opinion, be complete without one. The appropriations made by Congress doubtless were so made with particular reference to a literary department, for a large number of the members of Congress in voting, did not know what plan the States, to which this bounty was given, would adopt in the organization of a University.

This suggests an important question, which has not yet been definitely settled, and that is, what constitutes a University? It is maintained that the only one in existence, and which embraces all the departments constituting it such, is the German University. We have no such perfect organization in this country, and certainly none organized and sustained by the National Government. Lands, it is true, have been donated to different Western States, not only for the establishment of common schools, but also for the establishment and maintainance of Universities. Michigan, Iowa, and if we mistake not, Wisconsin, were favored by these appropriations. Then, if it were understood by Congress that these Universities were to embrace, as an integral part, a medical department, in that event, it indirectly aided in the establishment, and provided also for the maintainance of Medical Schools. But we are yet to be informed what is, or may be, the decision upon this question. If it was the intention of the National Legislature that a University should embrace the usual and ordinary departments constituting it such, then the benefits should be enjoyed, and then too, the *Peninsular Journal* is manifestly correct in calling Dr. Campbell's attention to the mistake he has made, and justified in raising its voice in the vindication of the Nation from the charge made before a foreign audience.

There are those in this State who have maintained that, to constitute our University, a literary department only is necessary. This opinion is entertained by some who claim intelligence and prominence, but who are troubled with certain proclivities for *pathies*. These would allow the medical profession to waste, wither, and perish, without a fountain from which to slake their thirst for knowledge.

But a number of the States, in their individual capacity, and from their own treasury, have magnanimously made liberal bestowments toward the organization and maintainance of their medical schools. Prominent among these is Louisiana, which made large appropriations to the old institution in New Orleans, and there is little doubt but that the same liberality will be extended to the young and promising school recently established. Ohio, too, aided one of her medical institutions, and we believe that some of those in the older States were also materially aided. Notwithstanding there are a few such instances, the greater number of our schools are individual enterprises. There is certainly too much apathy manifested toward medical institutions. There are no enterprises of a scientific character in which the Professors labor more, or make greater sacrifices than those connected with medical schools. There is no class of men, when educated, from whom so much is expected as the physician. They are not only under the necessity, in this country, of projecting institutions for the cultivation of medical science, by their own efforts, but afterward to sustain them by their own means. Not this alone, but they are compelled to smuggle their own material for dissection, and are liable to heavy penalties if they are found in pursuit of that which is indispensable for instruction. And yet they *must* be educated, *must* be learned, and at the same time, the government and people withhold their aid and the means of improvement. Like Draco, who wrote his laws in a hand so small and hung them so high as not to be read by the populace, yet he punished severely for every infraction of them.

It is not to be concealed that medical science is left, in the main, to struggle alone, comparatively unaided, and this is the more apparent, when we contrast our course in this respect, with that of the old countries. In this country, which is the land of progress, and where there is the most ample provision made for the education of the young, it is an approbrium upon us that scientific enterprises should be allowed to struggle, and very often to languish. For our part we rejoice that Dr. C. has mooted this subject, and mooted it, too, where he did. The reverberation has come over the waters, and it may bestir our authorities to increased action, and to adopt a new and more liberal policy toward medical institutions.

The guardians of the nation, in their desire for a complete system of education throughout the country, should not, when making appropriations for Universities, omit to specify a portion to the medical department. If left to the Legislatures, this interest may be neglected, because of certain prejudices growing out of prevalent *isms* and *schisms*, which would tend to divide. In the success of medical science, the nation has a deep interest, as the army and navy require the highest acquirements on the part of the medical staff in both.

Let our governments, both State and National, provide amply for the establishment of medical schools, and wipe away the stain which affixes to us as a nation, by the neglect which is exhibited by them ot a department in science, pre-eminent for its efficiency and usefulness.

---

☞ *The Medical Times*, of N. Y., has merged itself into the *New York Journal of Medicine*, and the *Louisville Review* and the *Philadelphia Medical Examiner* have been consolidated under the title of the *North American Medico-Chirurgical Review*, to be under the control of Profs. Gross & Richardson, formerly of Louisville, Ky., but now occupying chairs in different schools in Philadelphia. The *Louisville Review* was most ably conducted under the editorial charge of these gentlemen, as was made evident in the few numbers which made their appearance, and we look for, in the new publication, a valuable contributor to medical science. But the title is quite too long, friends.

☞ Dr. G. C. Weber, formerly of New York, takes the chair of Surgery in the Cleveland Medical College, heretofore so ably filled by Prof. Ackly.

☞ Dr. Brown-Sequard delivered three lectures in the Universal Medical College, N. Y., on his new topic connected with the physiology of the nervous system.

☞ The editor of the *Western Lancet*, Cincinnati, reposes much confidence in the styptic powers of the *Perchloride of iron*, as a styptic. During an operation, he says that an assistant followed the track of his knife with the perchloride, and the hemorrhage was immediately arrested.

☞ The Extract of Belladonna applied around the nipple and covering the Areolæ in the case of tumid breasts, has produced relief by arresting the lacteal secretion. Such is the result of the observation and experience of Dr. Goodlens, of the St. Thomas Hospital.

☞ M. Delpech called the attention of the French Academy, to a new disease appearing in the workmen of gum Caoutchouc, the symptoms of which are, digestive derangement, deficient intelligence, dullness, loss of memory, derangement of the nervous system, with complete impotence.

☞ Dr. Dixen, like a successful gladiator, flourishes his Scalpel monthly, instead of quarterly. Although modified in form, its edge still preserves its keenness. He cuts in his own peculiar way, but no one doubts the science of the dissection.

THE last number contained an article written by a member of the Faculty, stating the fact that the senior editor of this Journal was at that time suffering from disease, with little prospect of speedy relief. To those in whose minds that statement awakened an interest, he would say that he is "himself again," and actively engaged in the discharge of his duties, with better health than before the attack, and would thank all for the interest manifested for his well-being.

